# *Nature Communications* from the point of view of our very first authors

**DOI:** 10.1038/s41467-023-44470-x

**Published:** 2024-01-05

## Abstract

On the 12th of April 2010, *Nature Communications* published its first editorial and primary research articles. The topics of these first 11 papers represented the multidisciplinary nature of the journal: from DNA damage to optics alongside material science to energy and including polymer chemistry. We have spoken with the corresponding authors of some of these very first papers and asked them about their experience of publishing in this then new journal and how they see *Nature Communications* now.

**Figure:**
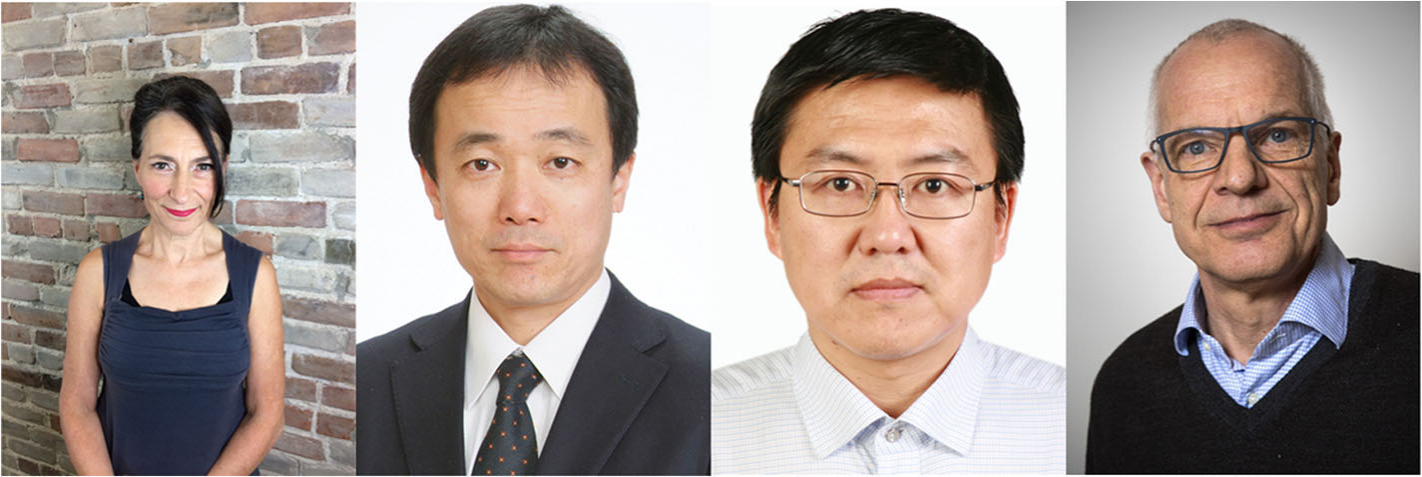
Prof. Jayne Yack (left) and her team study animal sensory systems and communication at Carleton University. Their paper on ‘The evolutionary origins of ritualized acoustic signals in caterpillars’ was accompanied by a video from our multimedia team. Prof. Masami Kamigaito (middle left) from Nagoya University and his team of polymer chemists reported on ‘Sequence-regulated vinyl copolymers by metal-catalysed step-growth radical polymerization’. Prof. Chuan-Feng Li’s (middle right) group performs research in quantum information and their paper published in *Nature Communications* on the 12th of April 2010 was an ‘Experimental investigation of classical and quantum correlations under decoherence’. Prof. Jens Stougaard (right) from Aarhus University and his collaborators published ‘The molecular network governing nodule organogenesis and infection in the model legume Lotus japonicus’. Jayne Yack, Masami Kamigaito, Chuan-Feng Li, Jens Stougaard respectively

Q1. What attracted you to publish your article with *Nature Communications*, at the time a new journal with an open access option? What would attract you now?

**Prof. Yack:** Back then we were interacting with established Nature branded journals and were not really aware of Nature Communications. We, however, followed editorial recommendation to submit to that journal. It seemed a bit risky at the time, as we knew nothing about the journal. However, now we certainly would submit directly to *Nature Communications*.

**Prof. Kamigaito:** What attracted us was that *Nature Communications* was a new platform issued by the then Nature Publishing Group covering all natural sciences, including chemistry and biology. Because we wanted to publish our article, which reported a new method to synthesize sequence-regulated vinyl polymers based on our original concept, in a multidisciplinary journal to attract not only polymer chemists but also other chemistry, materials, and biological scientists, I thought that *Nature Communications* was most suitable for this purpose. When we decided to submit our paper to *Nature Communications*, open access publication was optional. We did not choose open access for this paper because we did not fully grasp the importance and impact of it at that time. Now, I understand that open access is particularly important for an article that not only reports a new concept but also aims to attract scientists in other research fields, who would then be able to freely access the article without any charges and difficulties. There are now indeed many open access journals, even in specific research areas. Among them, *Nature Communications* still attracts us as one of the leading open access journals covering all natural sciences.

**Prof. Li**: Before *Nature Communications* came out, Nature-branded journals had a good reputation in the scientific community and had a great influence. At that time, we were submitting papers to these journals and we noticed the invitation letter for the launch of *Nature Communications*. Based on the trust in the *Nature* brand and an attempt for open access, we submitted our paper to *Nature Communications* which was accepted and published in the first issue in April 2010.

In the past 10 years, *Nature Communications* has been publishing more and more papers, and its influence has also grown. The scientific community has already recognized *Nature Communications*, and we are very happy to submit our high-quality work to *Nature Communications* regularly.

**Prof. Stougaard**: At the time *Nature Communications* was launched I was finalizing a manuscript presenting the relationship between a rather large number of genes comprising two interconnected plant developmental pathways. A complicated story that needed to be carefully introduced and well explained in order to be able to unfold the story and guide readers step by step. This added up to a relatively long main text outlining the background and rational, which together with the large body of results presented in figures and tables made the manuscript suitable for a journal providing ample space. In contrast to print journals with tight word and page limitation, *Nature Communications* offered just that. I thought the *Nature Communications* format strived to allow the space for main text and the corresponding figures and tables that would be required to advance the presentation. I also found the clickable links highlighting figures, tables and references a very attractive feature that I anticipated would be a great help for readers navigating between different parts of a publication. On top of this, *Nature Communications* was open access with a broad coverage of scientific areas, which I felt matched the importance of the science in the manuscript and which I support in general in order to make science available to society without delay.

Q2. How has your research progressed since then, and how important was this paper in advancing your field/your research?

**Prof. Stougaard**: The Madsen et al. 2010 paper in the first *Nature Communications* online release built a network model summarizing a decade of gene discovery and was very well received by the community. It connected previous results into a conceptual framework that advanced the field and provided a stepping stone for subsequent experiments. In the following 10 years the field has advanced substantially in both breadth and depth, however the paper is still well cited in the field of molecular genetics of symbiotic and plant-microbe interactions. Since this early publication in *Nature Communications* I have published several other papers in *Nature Communications* and I still appreciate the flexible journal format.

**Prof. Kamigaito:** In the submitted paper, we reported a new concept and a successful result for perfectly controlling the monomer sequence of vinyl polymers. We proposed a new strategy based on a combination of the synthesis and controlled polymerization of sequence-regulated oligomers. We reported that the oligomers were obtained by iterative single monomer additions of vinyl monomers using Kharasch or atom transfer radical addition and that they were polymerized via step-growth radical polymerization after introduction of a vinyl group and a carbon-chlorine bond at each terminal.

Our research has led and will continue to lead to the development of highly functionalized vinyl polymers with excellent properties based on the sequence regulation of natural biopolymers, such as polypeptides, that have completely regulated amino acid sequences. This paper therefore introduced a new research field, which is now our main focus, for sequence-regulated synthetic polymers.

**Prof. Li:** Our work (*Nature Communications* 1, 7 (2010)) on the experimental investigation of classical and quantum correlations under decoherence had been proved to be of broad interest. It has been cited more than 330 times on Google. We then extended the study of quantum correlation dynamics to the case without system-environment back-action, which was also published in Nature Communications (*Nature Communications* 4, 2851 (2013)) and has been cited more than 160 times on Google. We was invited to contribute a review (*Int. J. Mod. Phys.* B 27, 1345054 (2013)) on the special issue “Classical vs Quantum correlations in composite systems” edited by L. Amico, S. Bose, V. Korepin and V. Vedral. During the investigation, we have opened up the direction of experimentally studying Markovian and non-Markovian quantum dynamics. We designed versatile setups to control the non-Markovianity of the environment. One of the related work was also published in Nature Communications (*Nature Communications* 9, 3453 (2018)).

**Prof. Yack:** We have made great progress with our work on the evolution and function of vibratory behaviour in caterpillars. The paper in *Nature Communications* was very important in conveying our research to a broad audience. There was a lot of media interest and general interest from the broad scientific community.

Q3. Ten years on, what would you say the role of *Nature Communications* is in supporting your research community? What else can we do?

**Prof. Yack**: During the past 10 years we have seen several new journals in the *Nature* family, so it becomes difficult to know which ones to follow. I think that the impact and visibility of the *Nature* journals decreases when there are so many subdivisions. It is often difficult for an author to know which of the ‘general’ journals to choose, and impact factor and readership of course play important roles. You could reach out to authors in specific areas, perhaps send them links of published papers in the field, to show them that you are interested in this kind of research. This would encourage more readership, more submissions of papers in particular fields and show support for these communities.

**Prof. Li:** Over the past ten years, my group has published a total of 8 papers in *Nature Communications*. We thank *Nature Communications* for providing such a high-quality and efficient platform. During this process, my group has been growing.

*Nature Communications* is already a very mature and professional journal. In the future, we hope to have more work published in *Nature Communications*. I wish *Nature Communications* better and better.

**Prof. Kamigaito**: *Nature Communications* is now one of the most valuable platforms for presenting a new concept and unprecedented results in polymer chemistry for not only polymer chemists but also for all natural scientists. In particular, I hope that *Nature Communications* continues to publish excellent papers that disseminate novel concepts and attract many natural scientists from other research fields to the research being conducted in the field of polymer science.

Alternatively, since *Nature Communications* covers very broad fields, it would be nice to have the research fields categorized efficiently so that a scientist in a specific field can easily access field-specific papers and can quickly read about exciting developments in their specific field. In addition, this would be beneficial for providing scientists with opportunities for widening their scopes to other fields.

From the perspective of a polymer chemist, I hope that *Nature Communications* continues to contribute to the development of polymer chemistry in natural sciences by publishing high-quality polymer science papers.

**Prof. Stougaard:** It seems to me that the *Nature Communications* approach has been widely accepted in the plant science community, as documented by many important publications that find their way into the journal. I believe the journal’s approach to the reviewing process has contributed to this development. As an author and also as a reviewer I find the editorial handling of reviews and decisions made on the basis of the reviewer comments is largely fair and balanced. This is an important asset in a time when many journal editors shy away from restricting the referees from having ever expanding expectations to have all possible experimental approaches included in one manuscript and have the next years follow-up research performed before accepting a piece of research that in itself progresses the field. Manuscripts should be well-focused and present results that support the conclusions and should not be a platform for referees to ask for add on experiments that would not change the conclusions. Large technical expansion in the last decades have greatly expanded the possible approaches to a particular question but this should not be taken as a reason for requesting additional experimentation. My best advice to *Nature Communications* is to further develop and maintain a journal policy and clear instructions that encourage reviewers to be open minded and dissociate themselves from current beliefs. Reviewers should balance the impact, strength and weakness of a manuscript constructively, setting aside any personal bias or preferences.

